# Comparative genomic analysis of two ST320 *Streptococcus pneumoniae* isolates, representing serotypes 19A and 19F

**DOI:** 10.1186/s12863-023-01118-5

**Published:** 2023-04-10

**Authors:** Weronika Puzia, Jan Gawor, Robert Gromadka, Anna Skoczyńska, Ewa Sadowy

**Affiliations:** 1 Department of Epidemiology and Clinical Microbiology, National Medicines Institute, Warsaw, Poland; 2 DNA Sequencing and Synthesis Facility, Institute of Biochemistry and Biophysics PAS, Warsaw, Poland; 3 National Reference Centre for Bacterial Meningitis, National Medicines Institute, Warsaw, Poland; 4 Department of Molecular Microbiology, National Medicines Institute, Ul. Chelmska 30/34, 00-725, Warsaw, Poland

**Keywords:** *Streptococcus pneumoniae*, Serotype, Serotype switch, Complete genome, Recombination, Antimicrobial resistance, Tn*2010* transposon

## Abstract

**Background:**

*Streptococcus pneumoniae* (pneumococcus) represents an important human pathogen, responsible for respiratory and invasive infections in the community. The efficacy of polysaccharide conjugate vaccines formulated against pneumococci is reduced by the phenomenon of serotype replacement in population of this pathogen. The aim of the current study was to obtain and compare complete genomic sequences of two pneumococcal isolates, both belonging to ST320 but differing by the serotype.

**Results:**

Here, we report genomic sequences of two isolates of important human pathogen, *S. pneumoniae.* Genomic sequencing resulted in complete sequences of chromosomes of both isolates, 2,069,241 bp and 2,103,144 bp in size, and confirmed the presence of *cps* loci specific for serotypes 19A and 19F. The comparative analysis of these genomes revealed several instances of recombination, which involved not only *S. pneumoniae* but also presumably other streptococci as donors.

**Conclusions:**

We report the complete genomic sequences of two *S. pneumoniae* isolates of ST320 and serotypes 19A and 19F. The detailed comparative analysis of these genomes revealed the history of several recombination events, clustered in the region including the *cps* locus.

## Introduction

*Streptococcus pneumoniae* (pneumococcus) represents one of the leading human bacterial pathogen in community-acquired respiratory and invasive infections [1, 2]. Polysaccharide capsule, specifying the serotype constitutes a key virulence factor of pneumococcus [3]. The composition of capsule, determined by the *cps* locus shows a remarkable diversity in the population of this pathogen [4, 5] and represents a pivotal component of anti-pneumococcal non-conjugated and conjugated vaccines [6]. Introduction of the 7-valent polysaccharide conjugate vaccine (PCV7, against serotypes 4, 6B, 9 V, 14, 18C, 19F and 23F) into a mass vaccination of children resulted in not only decreased an incidence of invasive infections in this group but also contributed to a reduction of resistance levels to antimicrobials important for a therapy [7], due to the fact that limited number of pneumococcal serotypes was then in a significant part responsible for the appearance of multi-drug resistance (MDR) among pneumococci [8, 9]. Multilocus sequence typing (MLST) introduced into analyses of pneumococcal populations [10] played an important role in determining that circulation of certain pneumococcal epidemic clones greatly contributed to increasing the levels of MDR among pneumococci [11] before the PCV7 era. Such clones were identified by the Pneumococcal Molecular Epidemiology Network (PMEN) and named following their country of first isolation and main serotype associated with a given clone [12]. Introduction of the whole-genome sequencing (WGS) into microbiology opened entirely new possibilities for analyses of pneumococcal clones and their evolution [13].

Post-vaccine surveillance revealed a quick adaptation of *S. pneumoniae* to the selective pressure exerted by PCV7, resulting in serotype replacement [14], caused by an increased circulation of clones associated with non-vaccine serotypes (NVT) and changes of serotypes within established epidemic clones [15–17]. Such change, named a “serotype switch” is known to occur in pneumococcal populations thanks to a natural competence of these bacteria and involves an exchange of the *cps* locus, often with its adjacent sequences [18]. In the US, following the introduction of PCV7 an appearance of “vaccine escape recombinants” expressing non-vaccine serotype 19A instead of serotype 4 targeted by the PCV7 was observed in the clone of sequence type (ST) 695 [19]. These changes in pneumococcal population prompted introduction of two higher-valent vaccines, PCV10 (against PCV7 serotypes plus 1, 5 and 7F) and PCV13 (against PCV10 serotypes plus 3, 6A and 19A), into the market in 2009. These vaccines either replaced PCV7 or were introduced de novo into the national or local vaccination calendars. Serotype replacement by 19A post-PCV7 was observed in the US and several other countries due to both increases of prevalence of strains typically associated with 19A, such as e.g. the clonal complex CC199^19A^ as well by spread of clones that had undergone serotype switch such as CC695^19A^ mentioned above [16]. Such replacement by 19A, mostly multidrug resistant, has also been noticed in countries where PCV10 has been used [20, 21]. In turn, the use of PVC13 has greatly reduced the number of 19A infections and thus the level of antibiotic resistance [22, 23] although this beneficial effect may be reduced by emergence of resistance in other non-vaccine serotypes, e.g. in Spain [24].

In Poland, the National Reference Centre for Bacterial Meningitis (NRCBM, Warsaw) since 1997 constantly monitors and provides laboratory confirmation for invasive pneumococcal infections in all age groups. The PCV10 was introduced into the vaccination calendar in Poland in 2017 [25] but before all PCVs were available commercially and used on a voluntary basis. A gradual increase in the 19A serotype prevalence was observed in the 2010s [25, 26] and based on our preliminary data, isolates of ST320 were the most prevalent ones among 19A isolates in Poland before the vaccination era [27]. To improve our understanding of the phenomenon of exchange of *cps* and other genes among particular pneumococcal clones, we present the complete genomic sequences of two pneumococcal isolates, representing the same sequence type, ST320 as defined by the MLST scheme but demonstrating two different serotypes, 19A and 19F, obtained in Poland from invasive infections.

## Methods

### Bacterial isolates

The 3238/09 and 3641/15 isolates, obtained from patients’ blood in hospitals in Wejherowo (54.6147 N, 18.2450 E) and Kraków (50.0120 N, 20.0012 E) were received by the NRCBM as a part of routine surveillance activity mandated by the Ministry of Health. The study was conducted as part of continuous surveillance in accordance with the World Health Medical Association 1966 Declaration of Helsinki and the EU rules of Good Clinical Practice, thus ethical approval and informed consent were not required. Upon arrival to the NRCBM isolates were streaked on the Columbia agar with 5% sheep blood (CBA) (Becton, Dickinson and Company, Franklin Lakes, NJ) and incubated for 18–24 hours at 37 °C with 5% CO_2_. Re-identification of isolates was performed using classical microbiological methods, such as an evaluation of colony morphology, Gram staining and testing optochin susceptibility and deoxycholate solubility [28]. Isolates were stored in -80 °C in trypticase soy broth (TSB) (Becton, Dickinson and Company, Sparks, MD) with 40% horse serum and 15% glycerol until further analysis.

### Serotype determination and antimicrobial susceptibility testing

The quellung reaction with serotype-specific antisera (SSI Diagnostica, Hillerod, Denmark) was used for serotype determination, as previously described [28, 29]. The minimum inhibitory concentrations (MIC) of 18 antibiotics were determined by the broth microdilution method (BMD) “in house” and interpreted as recommended by the European Committee on Antimicrobial Susceptibility Testing (EUCAST) [30]. Susceptibility to erythromycin and clindamycin was additionally verified on the basis of the double disc test, and resistant isolates were assigned to specific phenotypes, including constitutive MLS_B_ (cMLS_B_), inducible MLS_B_ (iMLS_B_) and efflux-mediated resistance (M-phenotype). Multi drug-resistance (MDR) was defined as resistance to at least one agent from three or more antimicrobial groups. The quality control strain was *S. pneumoniae* ATCC 49619.

### DNA isolation, WGS, read assembly, MLST and rMLST

For genomic sequencing bacteria were grown on CBA for 11–13 hours and isolation of DNA was performed using the SDS/phenol method [31]. In brief, bacteria were collected from CBA plates by washing and resuspending in 2 ml TE buffer. Six hundred microliters of such suspension were taken for the DNA isolation procedure. DNA concentration was determined using the Qubit fluorometer (Thermo Fisher Scientific, Waltham, MA) and the quality of DNA preparations was evaluated by electrophoresis in 0.8% agarose (Prona Agarose, Burgos, Spain). Short-read sequencing was performed with the MiSeq instrument (Illumina Inc., San Diego, CA), using NEB Ultra II FS kit (New England Biolabs, Beverly, MA) for a library construction and MiSeq v3 600 cycle sequencing kit. Quality control and trimming was performed with the fastp software version 0.20.0 [32]. MLST and ribosomal MLST (rMLST) were performed following the established schemes [10, 33] and using the Internet-accessible databases https://pubmlst.org/organisms/streptococcus-pneumoniae and https://pubmlst.org/species-id, respectively (last accessed 1st June 2022) [34] to identify particular alleles and resulting STs and rSTs.

Long-read sequencing of both isolates was performed using the GridION instrument (Oxford Nanopore Technologies, Oxford, UK). Long read libraries were constructed using SQK-RBK004 kit and sequenced on R9.4.1 flowcell. Raw nanopore reads were basecalled and demultiplexed using guppy 4.2.2. Adaptor removal using porechop [35] and data quality filtering using NanoFilt [36] resulted in 35,962 and 46,547 reads, 265.15 and 560.43 Mbp of sequencing data with the N50 value of 11.8 kb and 28.6 kb, respectively for 3641/15 and 3238/09 isolates. Hybrid assembly of Illumina and Nanopore long reads was performed with the Unicycler version 0.4.6 software [37].

### Genomic data analysis

Annotation of assembled genomic sequences was performed with the NCBI PGAP version 5.3 [38]. The Geneious Prime v.2022.1.1 software (Biomatters, Auckland, New Zealand) was used for visualization of genomes and additional analyses. The Average Nucleotide Identity (ANI) was calculated using an online ANI calculator (http://www.ezbiocloud.net/tools/ani; last accessed 27th April 2022) [39]. Ribosomal sequence types (rSTs) were established using the rMLST [33] database (https://pubmlst.org/species-id; last accessed 25th March 2022) [34]. Antimicrobial resistance determinants were detected and localized in the genomic sequences using the ResFinder 3.0 [40] online service (https://cge.cbs.dtu.dk/services/ResFinder/; last accessed 25th March 2022). The presence of phages was determined by initial searches with PHASTER (https://phaster.ca/; last accessed 11th February 2022) [41] followed by manual analyses. Similarity of nucleotide sequences to these reported by others and potential functions of gene products were investigated by blastn and blastx searches, respectively, in GenBank (https://blast.ncbi.nlm.nih.gov/; last accessed 30th May 2022). Regions of recombination were identified with Gubbins [42] and visualized in Phandango [43]. Artemis Comparison Tool (ACT) [44] was used for sequence alignments and visualization. For all the software the default parameters were used. Gene localization is provided relative to the R6 genome of *S. pneumoniae* [45].

## Results and discussion

### Bacterial serotypes and antimicrobial susceptibility

The 3238/09 and 3641/15 isolates represented serotypes 19F and 19A, respectively. Of these two serotypes, 19F was included in the past in PCV7 and is present in two conjugate vaccines used currently (PCV10, PCV13) in mass vaccination, which significantly reduced its incidence. In contrast, 19A is targeted only by PCV13, which contributed to curbing 19A infections after their increase associated with the serotype replacement following PCV7/PCV10 use [20–23]. The 3238/09 and 3641/15 isolates were both multidrug resistant (MDR) demonstrating resistance to penicillin (MICs 8 and 4 mg/L, respectively), ampicillin (8 and 16 mg/L, respectively), amoxicillin (8 and 4 mg/L, respectively), cefuroxime (both 64 mg/L), cefotaxime (4 and 8 mg/L, respectively), cefepime (16 and 8 mg/L, respectively), erythromycin (both > 32 mg/L), clindamycin (both > 32 mg/L), tetracycline (8 and 16 mg/L, respectively) and trimethoprim-sulfamethoxazole (16 and 8 mg/L, respectively). They were susceptible to increased exposure to levofloxacin (both 1 mg/L) and moxifloxacin (both 0.12 mg/L), and susceptible to meropenem (1 and 0.5 mg/L, respectively), doxycycline (0.5 and 1 mg/L, respectively), linezolid (both 0.5 mg/L), chloramphenicol (4 and 2 mg/L, respectively), rifampicin (0.0075 and 0.03 mg/L, respectively) and vancomycin (0.5 and 0.25 mg/L, respectively). Except for the rifampicin (two dilutions), the isolates differed by no more than a single dilution in MIC values. The MDR phenotype is frequently observed worldwide for pneumococci representing serotypes 19A and 19F [46, 47]. In Poland, between 2011 and 2013 86.7 and 70.5% of invasive 19A and 19F pneumococci, respectively, were MDR while this phenotype characterized 21.6% invasive *S. pneumoniae* in general [26].

### Illumina sequencing, MLST and rMLST

Sequencing performed with Miseq resulted in 276,713 and 305,632 paired-end reads, respectively, corresponding to 142.39 Mbp and 164.73 Mbp of sequence for the 3238/09 and 3641/15 isolates, respectively. Both these isolates belonged to ST320 (Table 1). Among 805 isolates of ST320 reported to the PubMLST database for *S. pneumoniae* (date accessed 11th April 2022), the complete serotype was available for 789 isolates, among which serotype 19A and 19F were characteristic for 685 and 101 isolates, respectively. Both serotypes showed a global distribution and were observed in a similar time span (1998–2019 for 19A and 2000–2020 for 19F). ST320 is a double locus variant (DLV) of ST236, originally described as characteristic for the Taiwan^19F^-14 PMEN clone, associated with serotype 19F and nonsusceptible to penicillin, tetracycline and erythromycin but in contrast to our isolates sensitive to clindamycin [12, 48]. Both ST320 and ST236 are included in a large clonal complex named CC320/271 with either ST236 or ST271 (a single locus variant, SLV, of ST320) considered its likely ancestor [16, 49, 50]. In the recently introduced core genome MLST (cgMLST) scheme CC320/271 corresponds to GPSC1 [50]. The 3238/09 and 3641/15 isolates were associated with rST573 and rST572, respectively, differing by three loci (*rpsG, rpsI* and *rplM*) out of 53 rMLST loci [33].

**Table 1:** Summary of genome data for two ST320 isolates of *S. pneumoniae*

Isolate	Serotype	ST	rST	Genome size (bp)	%GC	Number of CDSs	Number of pseudo-genes	SRA accession number for Illumina reads	SRA accession number for Oxford Nanopore reads	GenBank accession number	Sequencing depth (x) ONT, Illumina
3238/09	19F	320	573	2,103,144	39.8	2089	124	SRR17689170	SRR17689169	CP091451	237x, 74x
3641/15	19A	320	572	2,069,241	39.9	2025	125	SRR17689168	SRR17689167	CP091450	123x, 88x

*ST*, Sequence type, *rST* Ribosomal sequence type, *CDSs* Coding DNA sequences, *SRA* Sequence Read Archive

### Complete genomes of 19A and 19F isolates and their features

The WGS of two analysed isolates yielded complete closed chromosomes (Table 1) with the ANI equal 99.69%. Both genomes showed the presence of four complete rRNA loci and 58 tRNA genes. The *cps* locus of the 3238/09 isolate of serotype 19F was in 99.98% identical to the *cps* of the Taiwan^19F^-14 isolate (CP000921.1) and belonged to the subtype 19F-I [51]. The *cps* locus of the 3641/15 isolate of serotype 19A was in 100% identical to its counterparts in some other members of CC320/271, such as the 19A-ST320_99–176 isolate from Korea (CP063829.1) and the SP61 isolate from Germany (CP018137.1) of ST2432, an SLV of ST320. The structure of this *cps* locus represented the subtype 19A-III [51]. Both isolates carried the identical intact loci determining biosynthesis of pilus-1 (P1) and pilus-2 (P2) types of pili. These structures are considered important for pneumococcal colonization and disease [52–54], yet only a minor part of *S. pneumoniae* population i.e. below 30% carries pili genes [54, 55]. The presence of both pili types is a characteristic feature of some CC320/271 isolates [53, 55].

Reduced susceptibility to penicillin and other β-lactams in pneumococci is associated with changes in some of so-called penicillin-binding proteins (PBPs), in particular Pbp1a, Pbp2b and Pbp2x [56]. While the *pbp1a* gene was the same in both isolates, their *pbp2b* and *pbp2x* genes demonstrated 95.0 and 97.5% identity, respectively, and this difference most likely resulted from recombination events (see below). The *pbp2b* in the 19F isolate was shared with a number of isolates belonging to CC320/271, such as NUBL-1080, RMV7, SP64, SP61, 19A-ST320_99–176 and TCH8431/19A (LC198130.1, OV904788.1, CP018138.1, CP018137.1, CP063829.1 and CP001993.1, respectively) and in the 19A isolate this gene was novel, with the closest hit (98.4% identity) to *pbp2b* of the URAspn6056 isolate of unknown serotype from Portugal (AM779405.1) [57]. The *pbp2x* in the 19F isolate was identical solely to *pbp2x* of the NUBL-1080 and RMV7 isolates mentioned above, and the 19A isolate harboured a novel gene, identical in 98.7% to *pbp2x* of Tw03–308 of 6B serotype (KC522447.1) [58].

Both isolates harboured the *tet*(M) tetracycline resistance gene, and *erm*(B) and *mef*(A) macrolide resistance genes, in concordance with the observed phenotypes. All three genes were located in the same genomic region and its analysis demonstrated the presence of the Tn*2010-*type transposon with the right terminus located in the counterpart of *spr1764* and the left terminus positioned upstream an ORF corresponding to *spr1775* of the R6 genome [45]. Tn*2010* is a 26.4 kb composite derivative of Tn*916* with insertions of *erm*(B) and *mega* elements [59, 60]. The observed localization of Tn*2010* is characteristic for several genomes of isolates associated with CC320/271 belonging to 19A and 19F serotypes [59, 60]. Tn*2010* showed 99.8% identity in the two isolates due to the presence of a unique 42-bp deletion in the putative replication initiation protein gene, corresponding to ORF20 in the original Tn*916*.

A single phage, 18,529 bp in size was located in the 3238/09 genome between the CDSs corresponding to *spr0003* and *spr0004* in the R6 genome. This phage occurred also in the same genetic localization in other genomes of pneumococci belonging to CC320/271 such as the ST556, RMV7, Taiwan19F-14, 19A-19,087, 19A-19,339, 19A-19,343, 19A-ST320_99–176 and TCH8431/19A isolates (GenBank accession numbers: CP003357.2, OV904788.1, CP035237.1, CP071916.1, CP071917.1, CP071918.1, CP063829.1 and CP001993.1, respectively) and was in 99.99% identical to the *Streptococcus* spp. satellite phage Javan759 [61].

No plasmids were found in any of the two isolates, which is in agreement with a very rare occurrence of plasmids in *S. pneumoniae* in general [62].

### Putative recombination events affecting genomes of 19A and 19F isolates

**Fig. 1:**
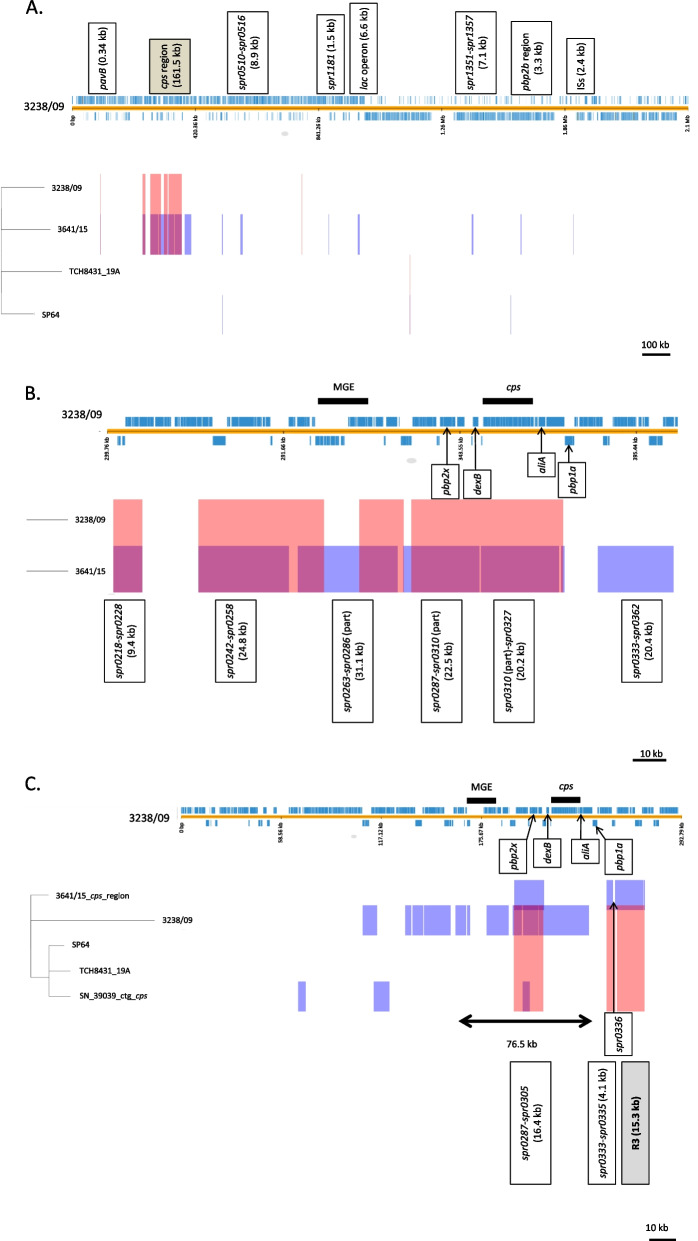
Recombination events affecting genomes of Polish invasive *S. pneumoniae* isolates of ST320 and serotypes 19A and 19F elucidated using Gubbins and visualized in PHANDANGO. The annotated 3238/09 genome used as reference, with short vertical blue lines of various thickness representing CDSs; phylogenetic trees on the left; pink bars, recombinations shared by clusters of isolates; violet bars, recombinations unique for isolates at terminal branches. **A** Analysis of the 3238/09 and 3641/15 genomes from this study and the complete SP64 and TCH8431-19A genomes of ST320 and serotype 19A from GenBank (CP018138.1 and CP001993.1, respectively). Recombination blocks, distinguishing the 3641/15 genome from the 3238/09 genome indicated above the reference genome; the approximately 161-kb *cps* region shadowed. **B** Details of the *cps* region from (**A**) depicted for the 3238/09 and 3641/15 genomes. The putative mobile genetic element (MGE) [49] and the *cps* operon marked by thick black bars; localization of *pbp2x, dexB, aliA, pbp1a* indicated with thin arrows; recombination blocks, distinguishing the 3641/15 genome from the 3238/09 genome indicated below. **C** Analysis of the approximately 0.3 Mb contig harbouring the *cps* operon from the SN39039 isolate [49] and the corresponding parts of the 3238/09, 3641/15, SP64 and TCH8431-19A genomes. MGE, *cps, pbp2x, dexB, aliA, pbp1a* indicated as in (**B**); the 76.5 kb recombining fragment, acquired by the SN39039 isolate [49] indicated by a thick double-headed arrow; recombination blocks upstream and downstream *cps* specific for the 3641/15 isolate described in the lower part of the figure, with the recombining segment R3 shadowed in grey

**Fig. 2:**
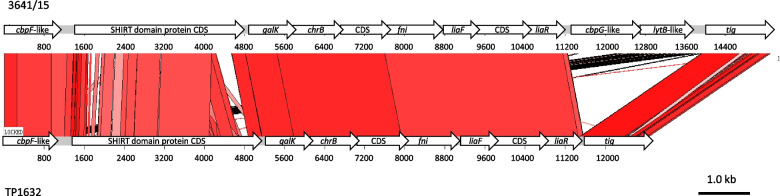
The 15.3 kb recombining segment R3 from 3641/15 isolate, compared to the corresponding part of the genome from the TP1632 isolate of *S. toyakuensis* sp. nov. using ACT. CDSs depicted as blue rectangles, homology blocks indicated in red

Analysis of two genomes revealed several instances of possible recombination events, especially in the region surrounding the *cps* locus (Fig. 1A). Two other complete genomes of *S. pneumoniae* of ST320 and serotype 19A, SP64 and TCH8431/19A available in GenBank (CP018138.1 and CP001993.1, respectively; date accessed 28th August 2020) were also included in these analyses. An internal recombination in the *pavB* gene (*spr0075*), whose product is a virulence factor involved in binding of host fibronectin and plasminogen [63], resulted in two different variants of *pavB* in 3238/09 and 3641/15. The difference between *pbp2b* of our two isolates, mentioned above was due to a local recombination event involving approximately the 3.3 kb segment carrying *pbp2b* (*spr1517*) and parts of the adjacent genes *recR* and *ybbH*. Other recombination events affected structural genes, e.g. the *metEF* genes and CDSs of unknown function in the region corresponding to *spr0510-spr0516*, *gdhA* (*spr1181*)*,* most of the *lac* operon (*spr1070-spr1077*) and the *glnHQP* genes, residing in the region corresponding to *spr1351-spr1357*. Recombination occurred also in a locus harbouring presumably functional and degenerate insertion sequences (*spr1701-spr1702*). The approximately 161-kb region including the *cps* operon was affected by a number of recombination events (Fig. 1B). Frequent recombinations in this area in CC320/271 were observed also by others [49] but the reason of this phenomenon is unclear. Apart from the exchange of the *cps* operon itself, five other recombination blocks, differentiating genomes of two Polish isolates were observed in this area. While one of these blocks contained the *pbp2x* gene, resulting in its different variants in the two isolates as described above, the *pbp1a* gene located downstream *cps* remained unaffected. In general, recombination blocks detected by analysis of the two genomes varied in size from 0.34 kb (*pavB*) to 31.1 kb (*spr0263-spr0286* in the *cps* region), in a good agreement with sizes of exchanged fragments observed for other vaccine escape recombinants [64]. It was proposed that at least four independent recombinations resulting in the change of original serotype 19F to 19A occurred in CC320/271 and one of these events, involving an approximately 76.5 kb region yielded a strain with ST320 and 19A serotype, represented by the SN39039 isolate [49]. In the case of other recombinant analysed in the same study, the 8312–05 isolate representing ST236^19A^, a potential donor of the *cps19A* genes belonged to ST199, however for SN39039 such donor could not be identified [49]. To investigate the relationship of SN39039 with Polish isolates, the approximately 0.3 Mb contig harbouring the *cps19A* operon from SN39039 was included in analysis with the corresponding parts of four previously analysed genomes (Fig. 1C). While the *cps19A* operon sequences in 3641/15 and SN39039 were identical, our analysis revealed three recombination blocks upstream and downstream *cps19A* distinguishing the 3641/15 isolate from SN39039. The 16.4-kb recombination block located upstream from 3641/15 showed 95.7% identity to the non-capsulated NT 100_58 strain [65] and of the two blocks identified downstream *cps19A,* the 4.1-kb region showed 96.4% identity to the KK1157 isolate (AP018044.1). Thus, the direct donor(s) of both these sequence blocks could not be determined. The 15.3-kb region marked R3 was most the most peculiar one, since it was most similar to a 12.9-kb fragment of the TP1632 genome of a newly proposed species, *Streptococcus toyakuensis* [66] (Fig. 2). These two fragments shared 82.0% identity. Two additional genes, presumably encoding CbpG-like and LytB-like proteins were present in R3 of the 3641/15 isolate. Both isolates in this part of the genome harboured a gene encoding a putative adhesin (pfam18655) containing Streptococcal High Identity Repeats in Tandem (SHIRT) domains reported in some species of viridians streptococci [67]. The deduced sequence of this protein was 1100 and 1224 amino acids long and harboured four and six SHIRT domains in 3641/15 and TP1632, respectively. To our knowledge, this is the first observation of the gene specifying an SHIRT domain protein in *S. pneumoniae*.

## Conclusions

In our study we report the complete genomic sequences of two isolates of *S. pneumoniae,* demonstrating serotypes 19A and 19F and belonging to ST320. The detailed comparative analysis of these genomes suggested several recombination events, particularly affecting the region including the *cps* locus, determining the biosynthesis of capsular polysaccharide, the major virulence factor of pneumococcus and the target of antipneumococcal vaccines. The complexity of recombination and gene acquisition events as well as lack of sequences of direct potential donors in GenBank precluded a complete reconstruction of evolutionary history of the *cps* region in the 3641/15 isolate, a presumable vaccine escape recombinant.

## Data Availability

This genome project is indexed at GenBank under BioProject accession number PRJNA799231. The complete genome sequences of *S. pneumoniae* 3641/15 and 3238/09 isolates are available at GenBank under accession numbers CP091450 and CP091451, respectively The raw sequencing data obtained during the study have been deposited in Sequence Read Archive (SRA) database under accession numbers SRR17689167, SRR17689168, SRR17689169 and SRR17689170.
